# Protective Effect of Jianpiyifei II Granule against Chronic Obstructive Pulmonary Disease via NF-*κ*B Signaling Pathway

**DOI:** 10.1155/2018/4265790

**Published:** 2018-08-05

**Authors:** Long Fan, Ruifeng Chen, Leng Li, Ziyao Liang, Xuhua Yu, Kai Huang, Shuomiao Yin, Lei Wu, Yuanbin Chen, Yinji Xu, Qi Wang, Lin Lin

**Affiliations:** ^1^The Second Clinical College of Guangzhou University of Chinese Medicine, Guangdong Provincial Hospital of Chinese Medicine, Guangzhou 510120, China; ^2^Institute of Clinical Pharmacology, Guangzhou University of Chinese Medicine, Guangzhou 510405, China

## Abstract

Jianpiyifei II granule (JPYF II) is an oriental herbal formula used clinically in China to treat chronic obstructive pulmonary disease (COPD). The aim of the present study was to investigate the anti-inflammatory and antioxidative activities of JPYF II in a mouse model of COPD induced by lipopolysaccharide (LPS) and cigarette smoke (CS) and in RAW264.7 cells stimulated with cigarette smoke extract (CSE). Mice were given LPS via intratracheal instillation on days 1 and 15 and exposed to CS generated from 4 cigarettes/day for 28 days. The mice were treated with 0.75, 1.5, or 3 g/kg/d JPYF II by intragastric administration in low, middle, and high dose groups, respectively, for two weeks. RAW264.7 cells were stimulated by CSE and treated with JPYF II at doses of 12.5, 25, or 50 *μ*g/mL. In the mouse model of LPS and CS-induced COPD, JPYF II decreased inflammatory cell counts in broncho alveolar lavage fluid (BALF), in addition to mRNA expression of proinflammatory cytokines and metalloproteinases (MMPs) in lung tissues. In addition, JPYF II elevated catalase (CAT) and glutathione peroxidase (GSH-Px) activities and reduced the levels of malondialdehyde (MDA) and I*κ*B*α* and p65 phosphorylation and inflammatory cell infiltration in the lung tissues. In RAW264.7 cells stimulated with CSE, JPYF II inhibited the mRNA levels of inflammatory mediators and the phosphorylation of I*κ*B*α* and p65. Our results suggest that JPYF II enhanced anti-inflammatory and antioxidative activities in a mouse model of COPD induced by LPS and CS and in RAW264.7 cells stimulated with CSE via inhibition of the NF-*κ*B pathway.

## 1. Introduction

Chronic obstructive pulmonary disease (COPD) describes a collection of chronic inflammatory diseases, including chronic bronchitis and emphysema. It is characterized by limited airflow which arises from an abnormal inflammatory response to inhaled particles and gases in the lungs [1], leading to alveolar and bronchial inflammation in susceptible patients. Cigarette smoke (CS) contains a complex proinflammatory mixture of chemical components which causes chronic inflammation in the airway and lung. Only 10%-20% of smokers develop clinically significant COPD, and additional exposure to workplace dust, chemicals, or occupational fumes may also result in this disease, but CS exposure is deemed to be a major risk factor related to the onset of COPD [2, 3] and almost 90% of COPD deaths are due to smoking [4].

COPD is characterized by the development of a complex inflammatory response in the lungs due to chronic exposure to irritants such as CS [5], consisting of the infiltration of inflammatory cells (e.g., macrophages, neutrophils, and T-lymphocytes) and increased expression of proinflammatory mediators (e.g., TNF-*α*, IL-6, and IL-1*β*) and matrix metalloproteinases (e.g., MMP-9 and MMP-12) [6]. Furthermore, the inflammatory process can be cyclically amplified as inflammatory cells are attracted from the circulation by the cytokines [7]. One important modulator of inflammation, NF-*κ*B, governs the generation of TNF-*α*, IL-6, and IL-1*β* [8]. Similarly, in the lipopolysaccharide- (LPS-) induced acute lung injury model, suppression of the NF-*κ*B pathway results in inhibition of lung neutrophil and cytokine generation [9, 10]. Furthermore, recent studies indicate that a number of MMPs may also contribute to airway remodeling [11, 12]. It is noteworthy that MMP-9, which is secreted primarily by macrophages and neutrophils, is considered a pivotal executor in an inflammatory response. In addition, it has been shown that emphysema induced by chronic CS exposure does not occur in MMP-12^−/−^ mice [13], indicating that MMP-12 can have a destructive role in COPD.

CS contains more than 10^15^ free radicals or oxidants and thousands of different chemical components per puff [14–17], which elevates the concentration of reactive oxygen species (ROS) produced enzymatically by epithelial and inflammatory cells in the lung. The production of additional ROS leads to the initiation of an inflammatory response through the activation of transcriptional factors such as activator protein (AP-1) and NF-*κ*B, in addition to gene expression of proinflammatory cytokines [17–19]. Therefore, oxidative stress also plays a key role in COPD and affects infiltration of surfactants to the alveoli, mitochondrial respiration, and remodeling of the extracellular matrix [17].

CS exposure is recognized as the major factor in the pathogenesis of COPD. In addition, as an important proinflammatory glycolipid component of the outer membrane of Gram-negative bacteria, LPS is often used in animal models of lung diseases, including COPD and acute lung injury [20–22]. Thus, a combination of LPS and CS has become the preferred* in vivo* irritant as it shortens the duration of exposure required for COPD to develop into an animal model and so enables deeper investigation of its pathological characteristics [21, 23–25]. Therefore, intratracheal administration of LPS and exposure to CS were combined to construct a mouse model of COPD in the present study. Moreover, CS activates innate immune cells such as macrophages, which play a key role in promoting inflammation and the secretion of inflammatory cytokines. It is known that their excessive activation has damaging effects and persistence of proinflammatory activity results in the development of chronic inflammation, such as COPD [26, 27]. Hence, exposure of the mouse macrophage cell line RAW264.7 to cigarette smoke extract (CSE) was also adopted in the present study.

Jianpiyifei II granule (JPYF II), an oriental herbal formula, consists of eight traditional Chinese medicines (TCMs) as shown in Table 1, including Radix Astragali, Rhizoma Cimicifugae, Radix Codonopsis, Rhizoma Atractylodis Macrocephalae, Radix Bupleuri, Herba Cynomorii, Fructus Viticis Negundinis, and Semen Persicae. Radix Astragali, Radix Codonopsis, and Rhizoma Atractylodis Macrocephalae are used in TCM to invigorate Qi [28], and Rhizoma Cimicifugae can be used to treat a collapse of the middle Qi [29]. Modern pharmacological studies suggest that they enhance natural immune function. Bu-Zhong-Yi-Qi-Tang (BT), a famous herbal formula used in China and Japan, whose formulation also includes Radix Astragali, Rhizoma Cimicifugae, Rhizoma Atractylodis Macrocephalae, and Radix Bupleuri, demonstrates immunomodulation and anti-inflammatory effects [30, 31]. Furthermore, Herba Cynomorii has been used in the treatment of kidney-yang deficiency, which can lead to asthma [32]. Fructus Viticis Negundinis has traditionally been used to treat chronic bronchitis and coughs [33]. Semen Persicae, which regulates lung Qi and removes stasis, is traditionally combined with other herbs to dissipate phlegm, relieve coughs, and treat asthma caused by the upward reversal of lung Qi [34]. Hence, JPYF II is formulated to treat COPD based on the traditional uses of the eight medicinal herbs described above using the principle of “Jun-Chen-Zuo-Shi” (“emperor-minister-adjuvant-courier”). Radix Astragali and Rhizoma Cimicifugae act as “Jun” to treat the main disease. Radix Codonopsis and Rhizoma Atractylodis Macrocephalae play the role of “Chen” to increase the function of “Jun”. Radix Bupleuri, Herba Cynomorii, Fructus Viticis Negundinis, and Semen Persicae treat the accompanying symptoms as “Zuo”.

JPYF II has been prescribed to treat COPD in Guangdong Provincial Hospital of Chinese Medicine for more than a decade. In a clinical study performed previously by the authors it was established that JPYF II was able to significantly increase walking distance over six minutes and decrease St. George's Respiratory Questionnaire (SGRQ) score in a treatment group of 178 patients, demonstrating that JPYF II had a clear therapeutic effect in stable stage COPD patients [35]. In addition, our previous studies demonstrated that JPYF II reduced airway resistance and intrathoracic pressure and attenuated ultrastructural damage to lung tissues in rats exposed to LPS and CS [36]. Furthermore, JPYF II protected the lung tissues of rats from exposure to LPS and CS by decreasing protein levels of TNF-*α*, IL-8, and TGF-*β*1, in addition to the MMP-9/TIMP-1 ratio [37]. However, the mechanisms of action of JPYF II or its protective effects against COPD are still not well-understood.

Therefore, the aim of the present study was to investigate the anti-inflammatory and antioxidative activities of JPYF II in a mouse model of COPD induced by LPS and CS and in RAW264.7 cells stimulated with CSE. The results demonstrate that JPYF II reduced pulmonary inflammation via suppression of the NF-*κ*B pathway and excessive oxidative stress, which indicated that it was the underlying mechanism of JPYF II in the treatment of COPD and provided a theoretical basis for future research and clinical application.

## 2. Materials and Methods

### 2.1. Plant Materials

JPYF II is a formulation of Radix Astragali, Rhizoma Cimicifugae, Radix Codonopsis, Rhizoma Atractylodis Macrocephalae, Radix Bupleuri, Herba Cynomorii, Fructus Viticis Negundinis, and Semen Persicae in a ratio of 3:1:3:1.5:1:1.5:1.5:1. All were purchased from Guangdong Provincial Hospital of Chinese Medicine and identified by their Department of TCMs. Additionally, specimens were deposited in the Second Clinical College of Guangzhou University of Chinese Medicine (Voucher Nos. 160717, 160718, 160719, 160720, 160721, 160722, 160723, and 160724). The medicinal herbs were powdered and extracted twice with 10 times their volume of boiling water for 90 minutes. Each water extract was filtered and dehydrated under vacuum and then residue was freeze-dried and stored in a refrigerator until required.

### 2.2. Chromatographic Analysis

Chromatographic analysis was performed using a Thermo Fisher Accela UPLC system (Thermo Fisher Scientific, San Jose, CA, USA) equipped with a quaternary pump solvent management system, an online degasser, a diode-array detector (DAD), a column compartment, and an autosampler using a Phenomenex UPLC Kinetex C_18_ column (2.1 × 100 mm, 1.7 *μ*m). The flow rate was maintained at 0.2 mL/min with a volume of injection of 3 *μ*L. The mobile phase consisted of an aqueous solution of 0.1% formic acid (A) and acetonitrile (B) with an elution gradient of 5%-25% B from 0 to 5 min, 25%-60% B from 5 to 28 min, 60%-90% B from 28 to 38 min, and 90% B between 38 and 42 min. Detection wavelengths were set at 214, 254, and 280 nm, with a column oven temperature set at 25°C.

### 2.3. Mass Conditions

Mass spectrometry (MS) was performed using a Thermo Fisher Accela LTQ Orbitrap XL hybrid mass spectrometer (Thermo Fisher Scientific, Bremen, Germany) equipped with an electrospray ionization (ESI) interface. The ESI source was set in positive ionization mode. Mass calibration was performed in accordance with the manufacturer's guidelines, using a standard solution mix of Ultramark, the tetrapeptide Met-Arg-Phe-Ala (MRFA) acetate salt, sodium taurocholate, and sodium dodecyl sulphate. The ionization and tube lens voltages were 4.2 kV and 86 V, respectively. Sheath and auxiliary gas (nitrogen) pressures were set at 20 and 5 units, respectively. Capillary temperature was set at 350°C. MS acquisition was set with a scan range of 150-1300* m*/*z* and a resolving power of 30000 for full-scan.

### 2.4. Reagents

Malondialdehyde (MDA), glutathione peroxidase (GSH-Px), and catalase (CAT) kits were purchased from Jiancheng Bioengineering Institute (Nanjing, China). I*κ*B*α* (#4812S), p-I*κ*B*α* (#9246S), and p-NF-*κ*Bp65 (#3033S) antibodies were obtained from Cell Signaling Technology (Danvers, USA). NF-*κ*Bp65 (#L0413) and *β*-actin (#G0213) antibodies were purchased from Santa Cruz Biotechnology (Texas, USA).

### 2.5. Animals

Female Balb/c mice (specifically pathogen-free and six to eight weeks old) were purchased from the Animal Supply Center of Guangdong Academy of Medical Science. All animals were housed under standard conditions in the Animal Center of Guangdong Provincial Academy of Chinese Medical Sciences at a temperature of 25±1°C and 55±5% humidity under a 12 h light/dark cycle with free access to water and food. All experimental procedures were approved by the Institutional Animal Care and Use Committee of Guangdong Provincial Academy of Chinese Medical Sciences.

### 2.6. Experiment Protocols

The mouse model of COPD was created by intratracheal administration of LPS and CS exposure daily. Briefly, mice were anesthetized with 4% pentobarbital and treated with LPS (0.2 *μ*g/*μ*L, 50 *μ*L) through intratracheal administration on days 1 and 15. Mice were exposed to CS generated from 4 cigarettes/day in batches of 10 in an 18 L Perspex chamber (40 cm × 25 cm × 18 cm) for 28 days. CS was collected in a 50-mL syringe over 10 s, mimicking the normal quantity of smoking inhalation and rate of cigarette combustion, and delivered four times per day at 11 AM, 12 noon, 1 PM, and 2 PM with 1 cigarette each. Control mice were exposed to air using a similar procedure. Filter-tipped DaQianMen cigarettes (manufactured by Shanghai Tobacco Group Co., Ltd, China) emitting 13 mg CO, 0.8 mg nicotine, and 11 mg tar per cigarette were used in this study. From the third week of CS exposure, mice in the positive group received 2 mg/kg/d dexamethasone and those in the treatment groups were treated with 0.75, 1.5, or 3 g/kg/d JPYF II by intragastric administration for two weeks. Mice in the control and model groups were intragastrically given an equal volume of saline.

### 2.7. Lung Homogenate

Lungs were harvested, rinsed thoroughly with ice-cold PBS, and homogenized with PBS. The homogenate was centrifuged at 12000 rpm for 15 min at 4°C, and the supernatant was collected into tubes and stored at −80°C until required for analysis.

### 2.8. Broncho Alveolar Lavage Fluid (BALF) Collection

Tracheostomies were performed and the tracheas were cannulated using a 24G blunt needle. 0.5 mL ice-cold PBS was injected and as much fluid as possible was withdrawn. The procedure was repeated twice. The cells were isolated by centrifugation, fixed, and then stained to distinguish the inflammatory cells in the BALF.

### 2.9. Cell Culture and 3-(4,5-Dimethylthiazol-2-yl)-2,5-diphenyl Tetrazolium Bromide (MTT) Assay

RAW264.7 cells, a mouse macrophage cell line, were cultured in Dulbecco's modified Eagle medium (DMEM) containing 100 IU/mL penicillin, 100 *μ*g/mL streptomycin, and 10% fetal bovine serum (FBS) in a humidified atmosphere of 5% CO_2_ at 37°C. Cells that reached 90% confluence were used for subsequent experiments. An MTT assay was performed to measure cell viability in response to JPYF II. RAW264.7 cells were treated with either JPYF II alone or in combination with CSE for 24 hours. Then MTT solution (10 *μ*L) was added for 4 hours at 37°C. Finally, 100 *μ*L of dimethyl sulfoxide (DMSO) was added to dissolve the resultant formazan crystals. Metabolic activity was determined from optical density at 490 nm using a microplate reader (PerkinElmer, Waltham, MA, USA).

### 2.10. Preparation of CSE

CSE was prepared in accordance with the procedure described by Wirtz and Schmidt [38]. Filter-tipped HongShuangXi cigarettes (manufactured by China Tobacco Guangdong Industrial Co., Ltd, China), emitting 13 mg CO, 1.2 mg nicotine, and 11 mg tar per cigarette, were used in this study. Briefly, a cigarette without filter was installed on a 50 mL syringe and completely combusted within 2 min. Smoke from two cigarettes was dissolved in 10 mL DMEM without serum. The final solution was adjusted to pH 7.4 and sterilized using a 0.22 *μ*m filter. Absorbance at 320 nm was measured using a spectrophotometer to standardize CSE preparation. The CSE solution was prepared prior to each experiment using exactly the same procedure, and the concentration of the final solution was defined as 100%. The CSE solution was diluted to the desired concentrations in accordance with the experiment.

### 2.11. Antioxidant Detection

Concentrations of MDA, GSH-Px, and CAT in the lung tissues were determined using appropriate detection kits according to the manufacturer's guidelines. Absorbance values were measured at 405, 412, and 532 nm, respectively.

### 2.12. Quantitative Real-Time PCR

Total RNA was isolated using NucleoZOL reagent (MN, Düren, Germany) from cultured cells or mouse lung tissues and reverse transcribed to cDNA using a reverse transcription kit (TaKaRa, Kusatsu, Japan). Real-time PCR expression analysis was performed on an ABI 7500 Real-Time PCR System (Applied Biosystems, Foster City, CA, USA) using SYBR® Premix Ex Taq™ (TaKaRa, Kusatsu, Japan). Primers used for PCR were as follows: IL-1*β*: 5′-GTCCTGTGTAATGAAAGACGGC-3′ (F), 5′-TGCTTGTGAGGTGCTGATGT-3′ (R); IL-6: 5′-ACAAAGCCAGAGTCCTTCAGAG-3′ (F), 5′-GAGCATTGGAAATTGGGGTAGG-3′ (R); TNF-*α*: 5′-ACGGCATGGATCTCAAAGAC-3′ (F), 5′-GGAGGTTGACTTTCTCCTGGTA-3′ (R); MMP-9: 5′-GCCCTGGAACTCACACGACA-3′ (F), 5′-TTGGAAACTCACACGCCAGAAG-3′ (R); MMP-12: 5′-ACACTACTGGAGGTATGATGTGAG-3′ (F), 5′-TGTTTTGGTGACACGACGGA-3′ (R); *β*-actin: 5′-GCTCCTAGCACCATGAAGATCA-3′ (F), 5′-AGGGTGTAAAACGCAGCTCA-3′ (R).

### 2.13. Western Blot Analysis

RAW264.7 cells and the homogenate of mouse lung tissues were lysed in ice-cold lysis buffer for 30 min. Total protein concentration in the lysate was measured using a bicinchoninic acid (BCA) assay. Normalized quantities of protein were separated using SDS-PAGE and then transferred to PVDF membranes which were blocked with 5% skim milk in Tris-buffered saline-Tween 20 (TBST) and incubated overnight at 4°C with primary antibody. After being washed with TBST, the membranes were incubated with secondary antibody for 2 h and the protein bands were visualized using a chemiluminescence detection system (Pierce, Rockford, IL, USA). Scanned images were quantified using Quantity One software (Bio-Rad, Hercules, CA, USA). GAPDH or *β*-actin was used as an internal control.

### 2.14. Histological Examination

Lung tissues were fixed using 4% (v/v) paraformaldehyde, paraffin-embedded, cut into 4 *μ*m thick slices, and then stained with hematoxylin and eosin solution (Sigma-Aldrich, CO, USA) for histopathological examination.

### 2.15. Statistical Analysis

Data from all the experiments were expressed as means ± standard deviation (SD). A one-way analysis of variance (ANOVA) with Tukey's multiple comparison test was applied to determine statistical significance.* P*-values < 0.05 were considered statistically significant.

## 3. Results

### 3.1. Identification of Phytochemicals in JPYF II Using HPLC-ESI-HRMS

**Figure 1:**
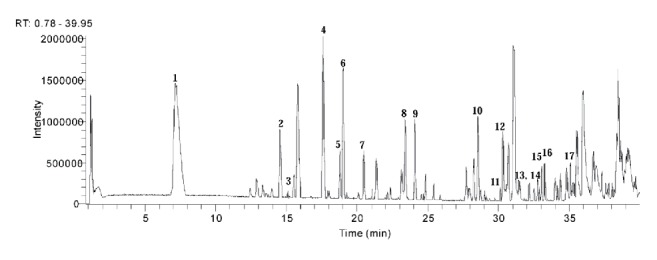
The total ion chromatogram (TIC) in positive ion (PI) mode of JPYF II.

By comparing the retention times and DAD-spectra of compounds previously reported or standard materials with the experimental compounds, the major components of JPYF II were identified using HPLC-ESI-HRMS.  Figure 1 displays the HPLC-ESI-HRMS total ion chromatogram (TIC). With reference to protonated molecular ion mass number, mass difference, and retention time compared with those of standards and literature data, a preliminary identification of peaks was performed, as listed in Table 2, with proposed compounds.

### 3.2. Effects of JPYF II on Body Weight of LPS and CS-Exposed Mice

**Figure 2:**
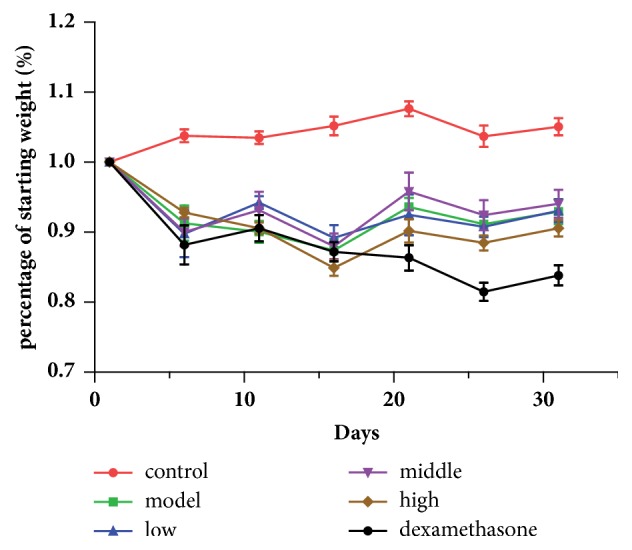
Effects of JPYF II on body weight of mice exposed to LPS and CS. Control: saline-treated mice; model: LPS and CS-exposed mice; low: JPYF II (0.75 g/kg/d) + LPS and CS-exposed mice; middle: JPYF II (1.5 g/kg/d) + LPS and CS-exposed mice; high: JPYF II (3 g/kg/d) + LPS and CS-exposed mice; dexamethasone: dexamethasone (2 mg/kg/d) + LPS and CS-exposed mice.

Mice exposed to LPS and CS lost weight compared with control mice (Figure 2). Treatment with either JPYF II or dexamethasone did not result in body weight returning to normal. However, those mice treated with JPYF II did not continue to lose weight unlike those treated with dexamethasone following stimulation with LPS and CS.

### 3.3. Effects of JPYF II on Inflammatory Cells in BALF

**Figure 3:**
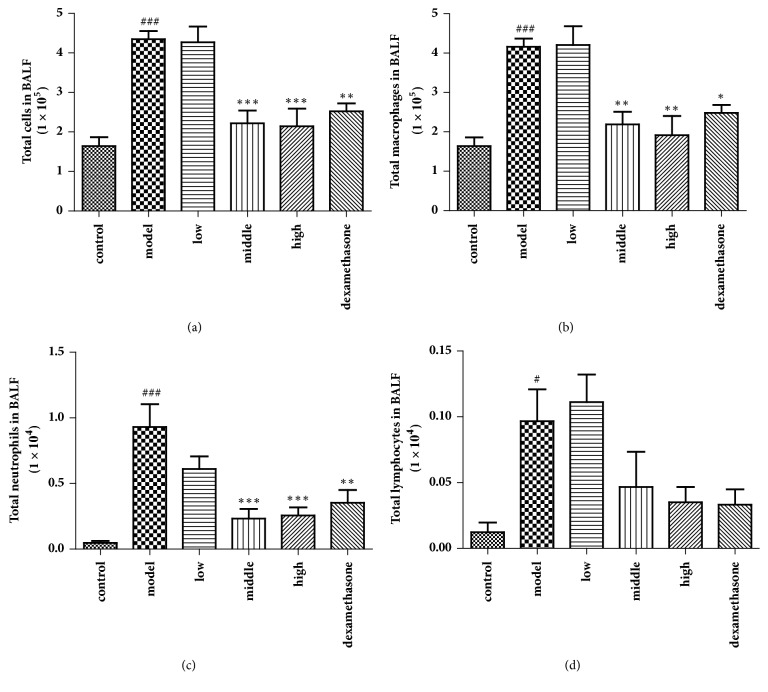
Effects of JPYF II on inflammatory cells in BALF. (a) Total cells in BALF, (b) total macrophages in BALF, (c) total neutrophils in BALF, and (d) total lymphocytes in BALF. Control: saline-treated mice; model: LPS and CS-exposed mice; low: JPYF II (0.75 g/kg/d) + LPS and CS-exposed mice; middle: JPYF II (1.5 g/kg/d) + LPS and CS-exposed mice; high: JPYF II (3 g/kg/d) + LPS and CS-exposed mice; dexamethasone: dexamethasone (2 mg/kg/d) + LPS and CS-exposed mice. Values are presented as means ± SD. ^#^*P* < 0.05 and ^###^*P* < 0.001 compared with control mice; ^*∗*^*P* < 0.05, ^*∗∗*^*P* < 0.01, and ^*∗∗∗*^*P* < 0.001 compared with LPS and CS-exposed mice.

The total and differential inflammatory cell numbers in BALF were quantified to determine the effects of JPYF II on inflammatory cells in LPS and CS-exposed mice. As Figure 3 demonstrates, JPYF II treatment significantly decreased the total inflammatory cell count in BALF, specifically the numbers of macrophages and neutrophils.

### 3.4. Effects of JPYF II on mRNA Expression of Proinflammatory Cytokines and MMPs in Lung Tissues

**Figure 4:**
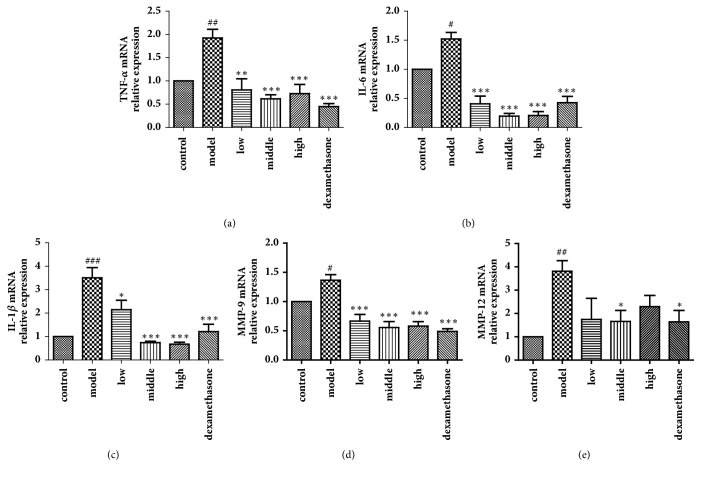
JPYF II decreased mRNA expression of proinflammatory cytokines and MMPs in lung tissues caused by LPS and CS exposure. (a) TNF-*α*, (b) IL-6, (c) IL-1*β*, (d) MMP-9, and (e) MMP-12. Control: saline-treated mice; model: LPS and CS-exposed mice; low: JPYF II (0.75 g/kg/d) + LPS and CS-exposed mice; middle: JPYF II (1.5 g/kg/d) + LPS and CS-exposed mice; high: JPYF II (3 g/kg/d) + LPS and CS-exposed mice; dexamethasone: dexamethasone (2 mg/kg/d) + LPS and CS-exposed mice. Values are presented as means ± SD. ^#^*P* < 0.05, ^##^*P* < 0.01, and ^###^*P* < 0.001 compared with control mice; ^*∗*^*P* < 0.05, ^*∗∗*^*P* < 0.01, and ^*∗∗∗*^*P* < 0.001 compared with LPS and CS-exposed mice.

As shown in Figure 4, LPS and CS-exposed mice demonstrated a notable elevation of the levels of the mRNA of TNF-*α*, IL-6, IL-1*β*, MMP-9, and MMP-12 in comparison to control mice. However, JPYF II-treated mice exhibited a significant decrease in TNF-*α*, IL-6, IL-1*β*, MMP-9, and MMP-12 mRNA levels in lung tissues compared with LPS and CS-exposed mice.

### 3.5. Effects of JPYF II on Antioxidant Activity

**Figure 5:**
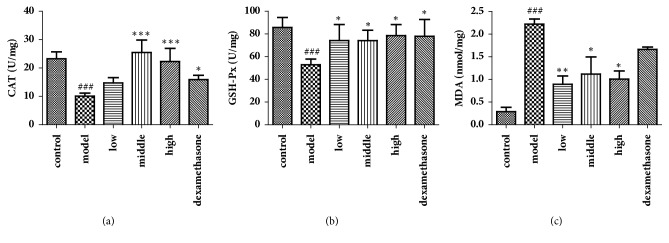
JPYF II increased the activities of CAT and GSH-Px, as well as decreased the level of MDA in lung tissues caused by LPS and CS exposure. (a) CAT, (b) GSH-Px, and (c) MDA. Control: saline-treated mice; model: LPS and CS-exposed mice; low: JPYF II (0.75 g/kg/d) + LPS and CS-exposed mice; middle: JPYF II (1.5 g/kg/d) + LPS and CS-exposed mice; high: JPYF II (3 g/kg/d) + LPS and CS-exposed mice; dexamethasone: dexamethasone (2 mg/kg/d) + LPS and CS-exposed mice. Values are presented as means ± SD. ^###^*P* < 0.001 compared with control mice; ^*∗*^*P* < 0.05, ^*∗∗*^*P* < 0.01, and ^*∗∗∗*^*P* < 0.001 compared with LPS and CS-exposed mice.

CAT and GSH-Px activities were reduced, and MDA levels were increased in the lung tissues of the LPS and CS-induced mice compared with those in control mice. However, treatment with JPYF II prevented the decrease in CAT and GSH-Px activities, and the elevation in MDA levels compared with those in LPS and CS-exposed mice. The results indicate that JPYF II was able to suppress excessive oxidative stress associated with COPD (Figure 5).

### 3.6. Expression of p-I*κ*B*α* and p-p65 in Lung Tissues

**Figure 6:**
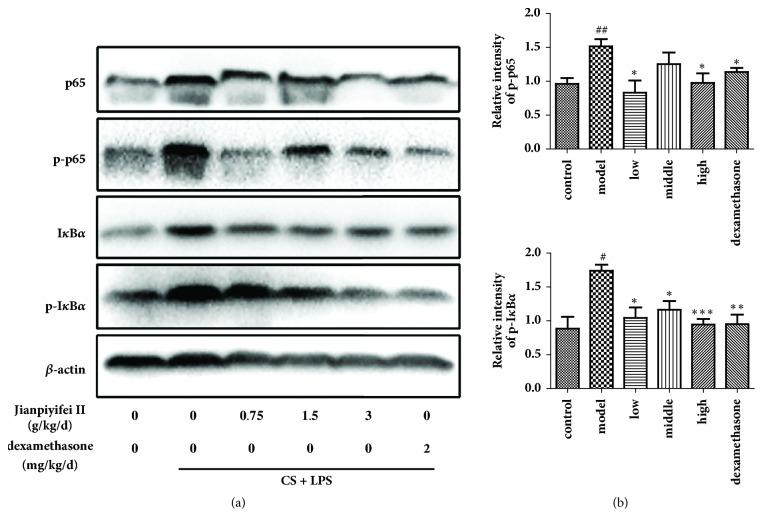
JPYF II reduced phosphorylations of I*κ*B*α* and NF-*κ*Bp65 in lung tissues caused by LPS and CS exposure. (a) Representative figure of protein expression and (b) quantitative analysis of protein expression. Control: saline-treated mice; model: LPS and CS-exposed mice; low: JPYF II (0.75 g/kg/d) + LPS and CS-exposed mice; middle: JPYF II (1.5 g/kg/d) + LPS and CS-exposed mice; high: JPYF II (3 g/kg/d) + LPS and CS-exposed mice; dexamethasone: dexamethasone (2 mg/kg/d) + LPS and CS-exposed mice. Values are presented as means ± SD. ^#^*P* < 0.05 and ^##^*P* < 0.01 compared with control mice; ^*∗*^*P* < 0.05, ^*∗∗*^*P* < 0.01, and ^*∗∗∗*^*P* < 0.001 compared with LPS and CS-exposed mice.

As Figure 6 demonstrates, phosphorylation of I*κ*B*α* and NF-*κ*Bp65 in lung tissues was clearly upregulated in LPS and CS-induced mice, which JPYF II treatment significantly reduced. The above results indicate that JPYF II suppressed the expression of p-I*κ*B*α*, which led to the inhibition of NF-*κ*B activation.

### 3.7. JPYF II Decreases Inflammatory Cell Infiltration Caused by Exposure to LPS and CS

**Figure 7:**
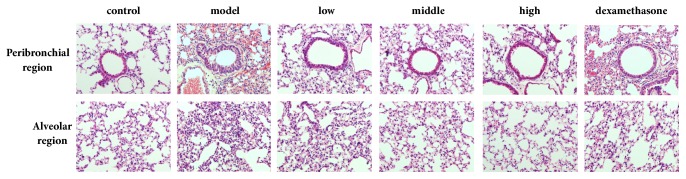
JPYF II inhibited inflammatory cell infiltration caused by LPS and CS exposure. Inflammatory infiltration in the peribronchial and alveolar regions was revealed by H & E staining. Control: saline-treated mice; model: LPS and CS-exposed mice; low: JPYF II (0.75 g/kg/d) + LPS and CS-exposed mice; middle: JPYF II (1.5 g/kg/d) + LPS and CS-exposed mice; high: JPYF II (3 g/kg/d) + LPS and CS-exposed mice; dexamethasone: dexamethasone (2 mg/kg/d) + LPS and CS-exposed mice.

As Figure 7 indicates, by comparison with control mice, a clear recruitment of inflammatory cells into the peribronchial and alveolar regions of the lung tissues of LPS and CS-exposed mice was observed. However, both JPYF II and dexamethasone-treated mice displayed reduced inflammatory cell infiltration in the bronchial airway compared with the LPS and CS-exposed mice.

### 3.8. Cell Viability of RAW264.7 Cells Exposed to CSE and Treated with JPYF II

**Figure 8:**
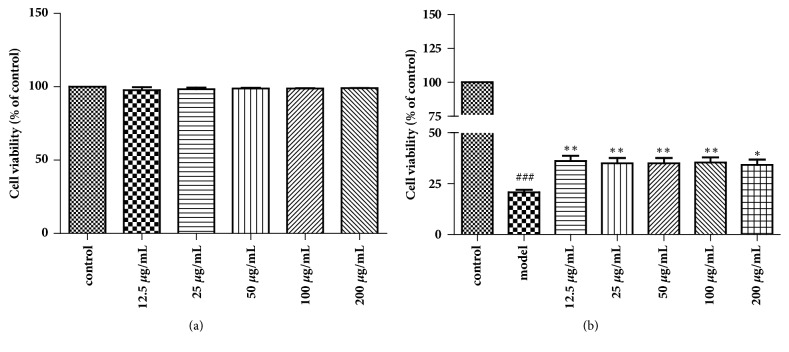
Cell viability was quantified by MTT assay. RAW264.7 cells were treated with various concentrations of (a) JPYF II or (b) in combination with CSE for 24 h. Values are presented as means ± SD. ^###^*P* < 0.001 compared with control group; ^*∗*^*P* < 0.05 and ^*∗∗*^*P* < 0.01 compared with LPS and CS-exposed mice.

An MTT assay was used to assess the viability of RAW264.7 cells exposed to various concentrations of CSE. The results reveal that the metabolism of cells was inhibited by CSE in a dose- and time-dependent manner (data not shown). A CSE concentration of 15% for 3 h was selected as the model because of its cytotoxic effects. The RAW264.7 cells were then treated with JPYF II (12.5, 25, 50, 100, and 200 *μ*g/mL) alone or in combination with 15% CSE for 24 h, demonstrating that JPYF II displayed no cytotoxic effects but enhanced the viability of the RAW264.7 cells (Figure 8).

### 3.9. Effects of JPYF II on the mRNA Expression of Proinflammatory Cytokines in RAW264.7 Cells Stimulated with CSE

**Figure 9:**
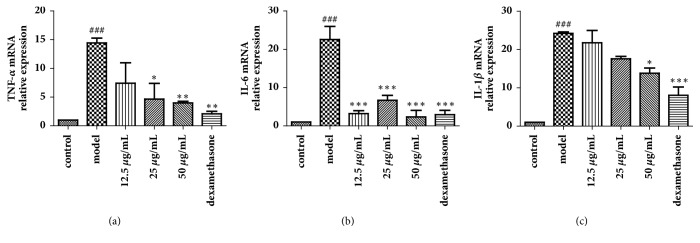
JPYF II reduced mRNA expression of proinflammatory cytokines in CSE-stimulated RAW264.7 cells. (a) TNF-*α*, (b) IL-6, and (c) IL-1*β*. control: RAW264.7 cells without any treatment; model: RAW264.7 cells induced by CSE; 12.5 *μ*g/mL: CSE-stimulated RAW264.7 cells treated with JPYF II (12.5 *μ*g/mL); 25 *μ*g/mL: CSE-stimulated RAW264.7 cells treated with JPYF II (25 *μ*g/mL); 50 *μ*g/mL: CSE-stimulated RAW264.7 cells treated with JPYF II (50 *μ*g/mL); dexamethasone: CSE-stimulated RAW264.7 cells treated with dexamethasone (50 *μ*g/mL). Values are presented as means ± SD. ^###^*P* < 0.001 compared with control group; ^*∗*^*P* < 0.05, ^*∗∗*^*P* < 0.01, and ^*∗∗∗*^*P* < 0.001 compared with model group.

The ability of JPYF II to suppress the mRNA levels of CSE-induced inflammatory cytokines (TNF-*α*, IL-6, and IL-1*β*) was measured. Their expression levels were elevated in RAW264.7 cells stimulated with CSE compared with nonstimulated cells, whereas the expression of TNF-*α*, IL-6, and IL-1*β* mRNA was suppressed in JPYF II-treated cells compared with CSE-stimulated cells (Figure 9).

### 3.10. Phosphorylation of I*κ*B*α* and p65 in RAW264.7 Cells Stimulated with CSE

**Figure 10:**
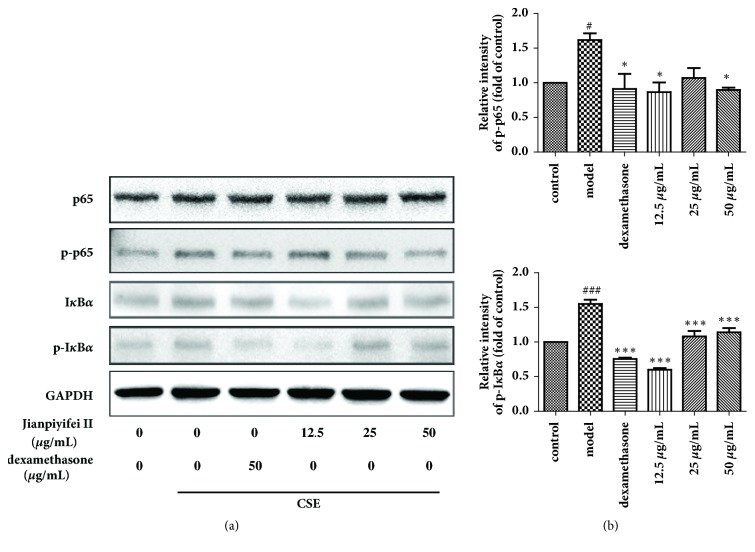
JPYF II inhibited the phosphorylation of I*κ*B*α* and NF-*κ*Bp65 expression in CSE-stimulated RAW264.7 cells. (a) Representative figure of protein expression and (b) quantitative analysis of protein expression. Control: RAW264.7 cells without any treatment; model: RAW264.7 cells induced by CSE; dexamethasone: CSE-stimulated RAW264.7 cells treated with dexamethasone (50 *μ*g/mL); 12.5 *μ*g/mL: CSE-stimulated RAW264.7 cells treated with JPYF II (12.5 *μ*g/mL); 25 *μ*g/mL: CSE-stimulated RAW264.7 cells treated with JPYF II (25 *μ*g/mL); 50 *μ*g/mL: CSE-stimulated RAW264.7 cells treated with JPYF II (50 *μ*g/mL). Values are presented as means ± SD. ^#^*P* < 0.05 and ^###^*P* < 0.001 compared with control group; ^*∗*^*P* < 0.05 and ^*∗∗∗*^*P* < 0.001 compared with model group.

As shown in Figure 10, the phosphorylation of I*κ*B*α* and p65 was clearly increased in RAW264.7 cells stimulated with CSE compared with nonstimulated cells, whereas JPYF II significantly suppressed the phosphorylation of I*κ*B*α* and p65 induced by the stimulation with CSE.

## 4. Discussion

In the present study, a mouse model of COPD induced by LPS and CS and RAW264.7 cells stimulated with CSE were used to evaluate the inhibitory effects of JPYF II on COPD. In the COPD model, JPYF II reduced the total and differential numbers of inflammatory cells in BALF. In addition, JPYF II attenuated inflammatory cell infiltration, which was evidenced by decreases in mRNA expression of proinflammatory cytokines and MMPs, and the phosphorylation of I*κ*B*α* and p65 in lung tissues. Furthermore, JPYF II increased CAT and GSH-Px activities, whilst decreasing MDA levels, indicating that JPYF II could inhibit excessive oxidative stress in the progression of COPD. In the RAW264.7 cells stimulated with CSE, JPYF II clearly suppressed the expression of proinflammatory mediators at the mRNA level, in addition to the phosphorylation of I*κ*B*α* and p65 expression.

COPD is characterized by reduced airflow and CS is believed to be the main risk factor in the progression of COPD. Exposure to CS elevates inflammatory cell counts, leading to constant airway remodeling. It also promotes the production of proinflammatory cytokines and proteases which aggravates COPD [39–42]. Proinflammatory mediators are associated with exacerbation of the inflammatory response in the development of COPD. In addition, previous studies have demonstrated that the concentrations of TNF-*α*, IL-6, and IL-1*β* increase significantly in human macrophages exposed to CSE and mice exposed to CS [43, 44]. This study demonstrated that treatment with JPYF II significantly suppressed excessive production of inflammatory cells and proinflammatory cytokines in LPS and CS-exposed mice. Moreover, JPYF II reduced the mRNA levels of TNF-*α*, IL-6, and IL-1*β* in RAW264.7 cells stimulated with CSE. These results indicate that JPYF II suppressed the inflammatory response associated with COPD.

MMPs are proteolytic enzymes which play a major role in breaking down components of the extracellular matrix (ECM) [45]. Previous studies have demonstrated that a number of MMPs, especially MMP-9, may also be associated with airway remodeling [11, 12], the migration of inflammatory cells into the lungs, and even the destruction of lung tissues [42]. Activated MMP-9 has been detected in the phlegm of the majority of COPD patients, but absent from healthy subjects, in addition to COPD patients possessing enhanced gelatinolytic activity related to MMP-9 in phlegm, confirming the importance of MMP-9 in COPD [46]. Additionally, compared to healthy subjects, the levels of MMP-12 in BALF from COPD patients were enhanced and the number of MMP-12-positive macrophages in both BALF and bronchial tissue sections of COPD patients was greater. These studies reveal that MMP-12 also plays an important role in COPD [47]. In this study, JPYF II treatment significantly suppressed the elevated levels of MMP-9 and 12 in mice exposed to LPS and CS. These results suggest that the ameliorative effect of JPYF II on COPD might also be associated with its inhibitory effects on MMPs.

Excessive ROS produced by epithelial and inflammatory cells stimulated by CS creates an oxidant/antioxidant imbalance leading to oxidative stress and induces lipid peroxidation causing serious damage to cellular and subcellular organelle membranes and function [48]. Antioxidant enzymes can either facilitate antioxidant reactions or directly decompose ROS [49]. GSH-Px and CAT play significant roles in peroxyl scavenging mechanisms and in maintaining the functional integration of cell membranes [50]. In addition, as the main product of lipid peroxidation, MDA is closely associated with pulmonary dysfunction and considered a biomarker in the assessment of lipid peroxidation [51]. Our investigation indicated that JPYF II administration could significantly increase CAT and GSH-Px activities and decrease MDA levels in lung tissues obtained from JPYF II-treated mice, demonstrating the antioxidation properties of JPYF II.

NF-*κ*B is normally present in an inactive form in the cytosol held by its repressor (I*κ*B) [43]. Activation of NF-*κ*B can be induced by proinflammatory stimuli which cause degradation of I*κ*B. CS is a potential stimulus that can induce the phosphorylation of NF-*κ*Bp65 and eventually activate NF-*κ*B [52]. In bronchial biopsies from smokers and in guinea pigs exposed to CS, NF-*κ*B has been shown to be significantly expressed, regulating the production of many inflammatory cytokines [53]. In the present study, phosphorylation of I*κ*B*α* and NF-*κ*Bp65 was meaningfully elevated in mice exposed to LPS and CS and RAW264.7 cells stimulated with CSE. However, JPYF II treatment inhibited activation of NF-*κ*B, indicating that JPYF II suppressed the inflammatory response caused by LPS and CS exposure or CSE stimulation, probably associated with downregulation of the NF-*κ*B pathway.

## 5. Conclusions

In summary, our study demonstrated that JPYF II provided a protective effect in LPS and CS-induced COPD mice through a reduction of the inflammatory response and oxidative stress. In addition, JPYF II suppressed the secretion of inflammatory cytokines in RAW264.7 cells stimulated with CSE. These effects might be related to suppression of the NF-*κ*B pathway. Hence, our study demonstrates that JPYF II has the potential to prevent COPD.

## Figures and Tables

**Table 1:** The components of JPYF II.

Herbal name	Botanical name	Family	Medicinal part
Radix Astragali	*Astragalus membranaceus* (Fisch.) Bunge	Leguminosae	Root
Rhizoma Cimicifugae	*Cimicifuga foetida* L.	Ranunculaceae	Rhizome
Radix Codonopsis	*Codonopsis pilosula* (Franch.) Nannf.	Campanulaceae	Root
Rhizoma Atractylodis Macrocephalae	*Atractylodes macrocephala* koidz.	Asteraceae	Rhizome
Radix Bupleuri	*Bupleurum chinense* DC.	Umbelliferae	Root
Herba Cynomorii	*Cynomorium songaricum* Rupr.	Cynomoriaceae	Whole plant
Fructus Viticis Negundinis	*Vitex negundo* L.	Verbenaceae	Fruit
Semen Persicae	*Prunus persica* (L.) Batsch	Rosaceae	Seed

**Table 2:** The mass numbers of LC/ESI-HRMS peaks and the identification results for the compounds of JPYF II.

No.	*t*_*R*_	[M+H]^+^	Formula	Identification	Source
	(min)	(mass error, ppm)			
1.	7.18	475.19174 (-1.36)	C_20_H_27_NO_11_	Amygdalin*∗*	Semen Persicae
2.	14.53	447.12805 (-1.17)	C_22_H_22_O_10_	Calycosin-7-*O*-*β*-d-glucoside*∗*	Radix Astragali
3.	15.11	414.23279 (-1.41)	C_20_H_28_O_8_	Lobetyolin*∗*	Radix Codonopsis
4.	17.60	357.13306 (-1.11)	C_20_H_20_O_6_	Vitedoin A	Fructus Viticis Negundinis
5.	18.79	357.13284 (-1.19)	C_20_H_20_O_6_	Isomer of vitedoin A	Fructus Viticis Negundinis
6.	19.01	533.12830 (-1.25)	C_25_H_24_O_13_	Calycosin-7-*O*-*β*-d-glycoside-6′′-*O*- acetate	Radix Astragali
7.	20.46	431.13315 (-1.18)	C_22_H_22_O_9_	Ononin*∗*	Radix Astragali
8.	23.42	285.07553 (-0.77)	C_16_H_12_O_5_	Calycosin*∗*	Radix Astragali
9.	24.09	517.13391 (-0.28)	C_25_H_24_O_12_	Formononetin-7-*O*-*β*-d-glycoside-6′′-*O*-acetate	Radix Astragali
10.	28.54	269.08057 (-0.99)	C_16_H_12_O_4_	Formononetin*∗*	Radix Astragali
11.	30.13	827.47821 (-0.65)	C_43_H_70_O_15_	Astragaloside II	Radix Astragali
12.	30.32	781.47327(0.02)	C_42_H_68_O_13_	Saikosaponin A	Radix Bupleuri
13.	32.50	231.13776 (-0.85)	C_15_H_18_O_2_	Atractylenolide I	Radix Codonopsis
					Rhizoma
					Atractylodis
					Macrocephalae
14.	32.50	249.14832 (-0.81)	C_15_H_20_O_3_	Atractylenolide III	Radix Codonopsis
					Rhizoma
					Atractylodis
					Macrocephalae
15.	33.02	869.48914 (-0.20)	C_45_H_72_O_16_	Astragaloside I/Isoastragaloside I	Radix Astragali
16.	33.28	781.47351 (-0.04)	C_42_H_68_O_13_	Saikosaponin D	Radix Bupleuri
17.	35.60	233.15353 (-0.33)	C_15_H_20_O_2_	Atractylenolide II	Radix Codonopsis
					Rhizoma
					Atractylodis
					Macrocephalae

*∗*Compared with standard compound.

## Data Availability

The data used to support the findings of this study are available from the corresponding author upon request.
